# AAV2-Mediated Subretinal Gene Transfer of mIL-27p28 Attenuates Experimental Autoimmune Uveoretinitis in Mice

**DOI:** 10.1371/journal.pone.0037773

**Published:** 2012-05-22

**Authors:** Ju Shao, Lichun Tian, Bo Lei, Lin Wei, Yan Yang, Aize Kijlstra, Peizeng Yang

**Affiliations:** 1 Chongqing Key Laboratory of Ophthalmology, Chongqing Eye Institute, Chongqing Medical University, The First Affiliated Hospital, Chongqing, People’s Republic of China; 2 Eye Research Institute Maastricht, Department of Ophthalmology, University Hospital Maastricht, Maastricht, The Netherlands; National Institute of Dental and Craniofacial Research, United States of America

## Abstract

**Background:**

Advances in gene transfer techniques have provided long-term, safe and stable transduction of retinal cells following subretinal injection with adeno-associated viral (AAV) vectors. In this study we investigated whether subretinal injection of AAV2-murine IL-27p28 vector was effective in inhibiting experimental autoimmune uveoretinitis (EAU) induced in B10RIII mice.

**Methodology/Principal Findings:**

An AAV2 vector encoding the murine IL-27p28 gene (rAAV2-CMV-mIL-27p28) was prepared and subretinally injected into B10RIII mice (4.35×10^8^ vector genome (v.g.)). AAV2 vector mediating green fluorescent protein (rAAV2-CMV-GFP) served as a control (5×10^8^ v.g.). The concentration of mIL-27p28 in homogenized eyes and serum was assayed by enzyme linked immunosorbent assay (ELISA) after subretinal injection. Human IRBP_161–180_ peptide and Complete Freund’s Adjuvant were injected into mice receiving either the rAAV2-CMV-mIL-27p28 or rAAV2-CMV-GFP vector. EAU was evaluated clinically and pathologically. The level of IL-17 and IL-10 in homogenized eyes was measured on day 12 and day 21 following immunization. Delayed type hypersensitivity (DTH) and IRBP_161–180_–specific proliferation of lymphocytes from the spleen and lymph nodes were assayed to examine the influence of the subretinal delivery of rAAV2-CMV-mIL-27p28 on the systemic immune response. IL-27p28 was detectable by ELISA within the eyes from two weeks following subretinal injection of the rAAV2-CMV-mIL-27p28 vector and showed a sustained high expression from day 14 to 9 months with a highest expression at 5 months. Subretinal injection of the vector significantly attenuated the severity of EAU disease clinically and pathologically in association with a significantly decreased IL-17 expression and an increased IL-10 expression. The IL-27p28 vector did not affect the systemic immune response, as determined by DTH and IRBP_161–180_–specific lymphocyte proliferation.

**Conclusions/Significance:**

A high and stable expression of IL-27p28 was observed for at least 9 months following subretinal delivery of rAAV2-CMV-mIL-27p28. The amelioration of EAU disease severity was associated with a decreased IL-17 expression and an increased IL-10 expression.

## Introduction

Uveitis is a sight-threatening inflammatory disease that mostly affects young adults and remains one of the important causes of visual impairment [1]–[3]. Corticosteroids and other immunosuppressive drugs have been widely used for its treatment [4]–[5]. However, these drugs may cause a number of side effects that can potentially be life-threatening.

Interleukin (IL)-27 is a recently identified member of the IL-6/IL-12 family. It is a heterodimeric cytokine consisting of 2 subunits, Epstein-Barr-induced gene 3 (EBI3) and IL-27p28 [6]. It has been shown to play an important role as an inhibitor of both Th1 and Th17 responses and may thus control autoimmune mediated inflammation [7]–[11]. Recent studies have shown that systemic administration of exogenous IL-27 significantly inhibits the activity of experimental autoimmune encephalomyelitis (EAE), EAU and collagen-induced arthritis (CIA) in animal models [12]–[14]. In addition, endogenous production of IL-27 by retinal cells has been shown to inhibit the intraocular inflammation in EAU [15]. IL-27p28 and EBI3 are secreted independently, and both subunits are not expressed by the same cells [16]–[17]. The finding that the IL-27p28 can be secreted in the absence of EBI3 suggests that this subunit may have an independent function. Purified IL-27p28, like heterodimeric IL-27, was capable of suppressing IL-17 production by CD4^+^ T cells in vitro [18]. Expression of IL-27p28 has been shown to result in a modest delay in the onset and severity of EAE [19].

Remarkable success of gene therapy has been achieved in human and animal models for various retinal diseases [20]–[23]. The eye is an organ particularly suitable for gene therapy due to the following reasons [24]. Various routes of delivery can be used to target layers in the eye under direct vision and the application of AAV vector leads to a long-term and stable gene transfer instead of having to use repeated injections and high dose administration of recombinant cytokines. In addition, single and low dose administration of AAV vector and the ocular immune privilege may reduce the immunogenicity. The elicited minimal host immune response following intraocular injection does not create retinal toxicity nor does it inhibit transgene expression [25]–[26].

Although AAV-mediated clinical gene therapy trials for diseases involving different organs have been approved (http://www.wiley.com/legacy/wileychi/genmed/clinical/), only few studies have been performed in the EAU model [27]–[28]. In this study we injected rAAV2-CMV-mIL-27p28 subretinally into the B10RIII mice, and longitudinally evaluated the expression of IL-27p28 within the eye for a time period of 9 months. The results showed a high and stable IL-27p28 expression within the eye and significant inhibitory effects on EAU. Moreover, this inhibitory effect was associated with a decreased IL-17 expression and an up-regulated IL-10 expression within the eye.

## Results

### IL-27p28 Expression following Subretinal Injection of rAAV2-CMV-mIL-27p28 Vector

**Figure 1:**
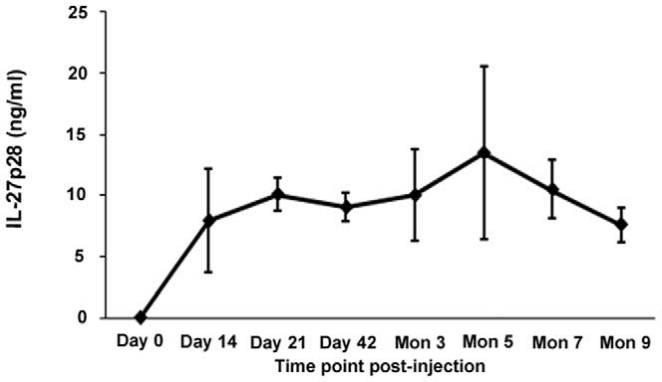
The expression of transgene following subretinal injection of rAAV2-CMV-mIL-27p28. The intraocular IL-27p28 levels at various time points show that IL-27p28 expression is stable over 90 days, its expression reaches a peak on day 150 and remains detectable until 9 months. Results are expressed as the mean ± standard deviation. (n = 3).

A group of mice was injected subretinally with rAAV2-CMV-mIL-27p28 (4.35×108 v.g.). The IL-27p28 level in supernatants from homogenized eyes obtained at different time points was assayed by an IL-27p28 specific ELISA. The result showed that IL-27p28 expression was detectable on day 14 (mean 7.916 ng/ml) and increased on day 21 (mean 10.076 ng/ml). A high and stable level of IL-27p28 expression was observed from day 42 (mean 9.011 ng/ml) up to day 150 (mean 13.472 ng/ml) and was followed by a decreased expression nine months after injection (the last detection point) (Figure 1). IL-27p28 could not be detected in the contralateral uninjected eyes or in rAAV2-CMV-GFP injected eyes at the various time points included in our study. It could also not be detected in undiluted serum over time.

### The Effect of rAAV2-CMV-mIL-27p28 on EAU

All B10RIII mice immunized with 50 µg human IRBP_161–180_ peptide emulsified in CFA showed a clinically visible EAU. The inflammation appeared on day 8 or 9 after immunization, reached its peak on day 12 and was followed by a gradual regression. No inflammation was observed in mice receiving CFA alone.

To test the effect of rAAV2-CMV-mIL-27p28 on EAU, it was subretinally injected into the right eyes of mice. rAAV2-CMV-GFP (5×10^8^ v.g.) control vector was injected into the contralateral eye of the same animal and served as an internal control [27]. Another group of mice only receiving a subretinal injection of PBS served as a separate control. All mice were subsequently immunized with IRBP_161–180_ peptide emulsified in CFA 3 weeks after subretinal injection.

**Figure 2:**
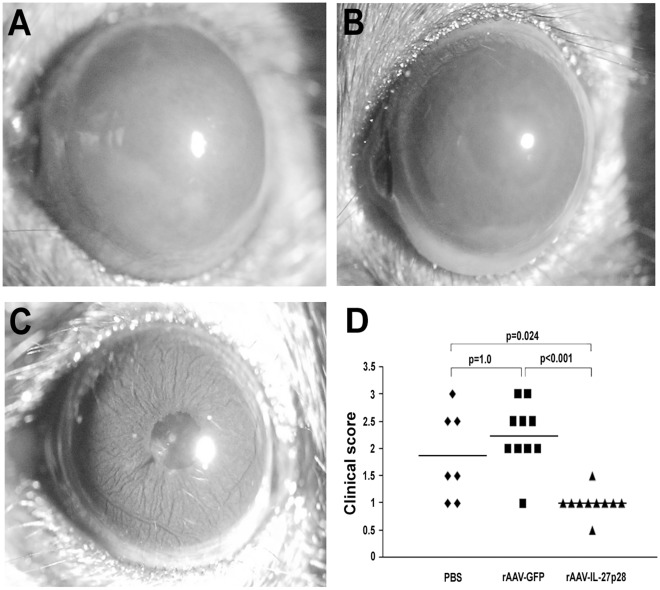
Clinical evaluation of EAU activity. rAAV2-CMV-mIL-27p28 was subretinally injected into the eye, PBS and rAAV2-CMV-GFP were used as controls. Three weeks after injection, mice were immunized to induce EAU with IRBP_161–180_ peptide and slit lamp microscopy was used to examine ocular inflammation. Images show significantly more severe inflammation in the PBS (A) and rAAV2-CMV-GFP injected eyes (B) as compared with the rAAV2-CMV-mIL-27p28 treated eye (C). Clinical scoring on day 12 after immunization (D) showed that the PBS injected eyes had a score of 1.86 (±0.8) and the rAAV2-CMV-GFP injected eyes reached a mean clinical score of 2.25 (±0.59), whereas the score of rAAV2-CMV-mIL-27p28 treated eyes was 1 (±0.24, p = 0.024, p<0.001, respectively). The scores of three chosen eye pictures are 3, 3 and 0.5, respectively. Each point represents an individual eye, the average scores of each group are denoted by the horizontal lines.

**Figure 3:**
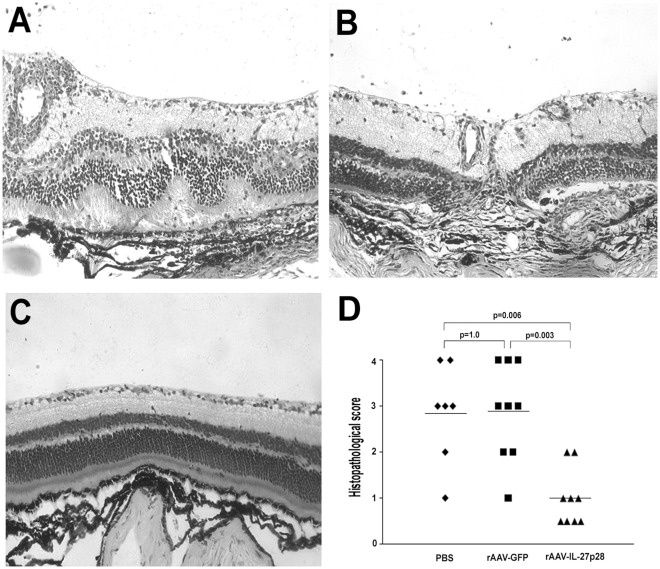
Histological examination on day 14 of EAU. Images of histological analysis show severe retinal folding, destruction, damage of the photoreceptor layer and massive inflammatory cell infiltration in the retina and the choroid, as well as intensive vasculitis in PBS injected eyes (A), moderate infiltration, moderate photoreceptor cell damage and medium-sized granulomas in rAAV2-CMV-GFP injected eyes (B). However, a minor infiltration of cells was observed in the choroid in rAAV2-CMV-mIL-27p28 treated eye (C). (hematoxylin eosin staining, magnification ×200) Comparing PBS injected and rAAV2-CMV-GFP injected eyes with rAAV2-CMV-mIL-27p28 treated eyes showed a reduced EAU histological grade (D, p = 0.006, p = 0.003, respectively). The scores of the three chosen eye section are 4, 3 and 0.5, respectively. Each point is the score of an individual eye, the mean scores of each group are denoted by the horizontal lines.

Obvious inflammatory reaction, as evidenced by conjunctival hyperemia, ciliary injection, corneal edema, aqueous cells and posterior synechiae, was observed in PBS or rAAV2-CMV-GFP injected eyes (Figure 2A, B). However, in rAAV2-CMV-mIL-27p28 treated eyes, a significantly decreased inflammatory reaction (Figure 2C) was observed as compared to PBS or rAAV2-CMV-GFP injected controls (p = 0.024, p<0.001, respectively) (Figure 2D). Histological examination on day 14 showed a severe intraocular inflammation as evidenced by massive infiltration of inflammatory cells throughout all retinal layers and the choroid, intensive retinal vasculitis and destruction of the retinal architecture, as well as photoreceptor damage in the PBS injected eyes and rAAV2-CMV-GFP injected control eyes (Figure 3A, B). However, only scattered infiltration of inflammatory cells into the retina and choroid was observed in rAAV2-CMV-mIL-27p28 treated eyes (Figure 3C). Histopathological grading showed that PBS injected eyes (EAU grade, 3.08±0.66) and rAAV2-CMV-GFP injected eyes (EAU grade, 2.89±1.05) had much more severe inflammation as compared with the rAAV2-CMV-mIL-27p28 treated eyes (EAU grade, 1±0.61) (p = 0.006, p = 0.003, respectively) (Figure 3D).

### The Effect of rAAV2-CMV-mIL-27p28 on the Level of IL-17 and IL-10 within the Eyes

**Figure 4:**
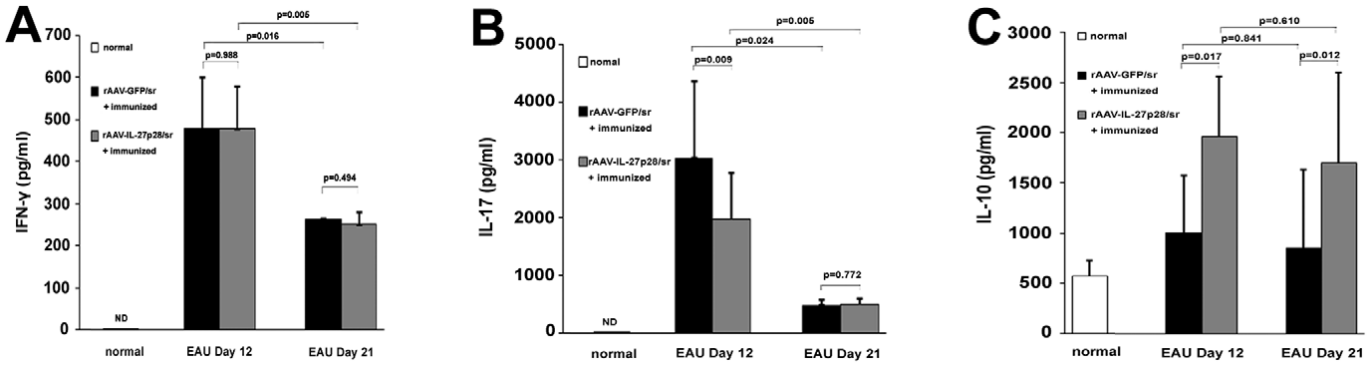
Intraocular cytokines immunoassay on day 12 and day 21. Normal mice and mice receiving a subretinal injection and a subsequent IRBP immunization were sacrificed on day 12 and day 21 post-immunization. The ocular cytokine levels were assayed by ELISA. IFN-γ (A), IL-17 (B) and IL-10 (C) levels in rAAV2-CMV-mIL-27p28 treated eyes compared with rAAV2-CMV-GFP treated eyes (p values 0.009 and 0.772 were obtained by a paired T test. The p value 0.024 was calculated using the Mann-Whitney U test). Results are presented as mean ± standard deviation. ND = not detected. (n = 5).

In order to evaluate whether the inhibitory effect of rAAV2-CMV-mIL-27p28 on EAU activity was associated with modulation of relevant cytokines, we measured the expression of IFN-γ, IL-17 and IL-10 within the eyes (figure 4A, B, C). The result showed that there was no detectable expression of IFN-γ and IL-17 within the normal eyes. An increased expression of these two cytokines was observed following immunization with IRBP_161–180_ peptide and CFA. A significantly higher expression for both IFN-γ and IL-17 was noted on day 12 as compared to day 21. However there was no difference concerning IL-10 expression between day 12 and 21. Subretinal injection of rAAV2-CMV-mIL-27p28 was associated with a significantly lower IL-17 expression on day 12 as compared with rAAV2-CMV-GFP treated groups. On the other hand we observed an increased IL-10 expression on both day 12 and 21 in rAAV2-CMV-mIL-27p28 injected eyes. No effect was found on IFN-γ expression between day 12 or day 21 when comparing GFP with IL-27p28 vector injected eyes.

### Effects of Subretinal Injection of rAAV2-CMV-mIL-27p28 on the Systemic Immune Response

**Figure 5:**
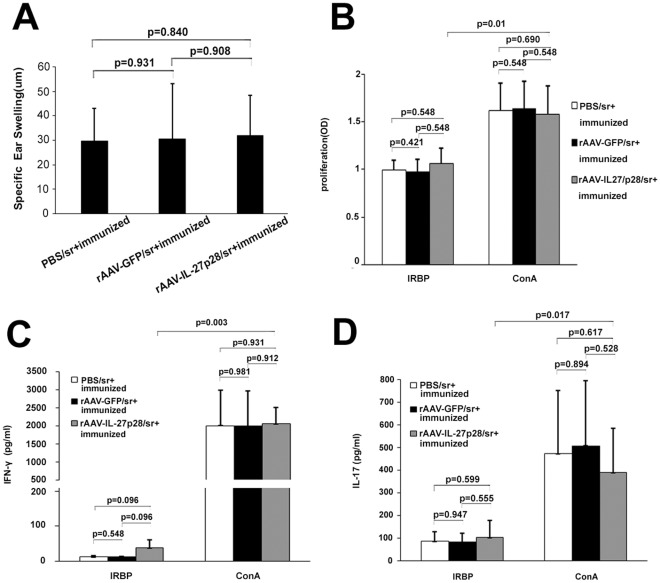
Systemic immune responses following subretinal IL-27p28 gene transfer. In vivo DTH (A), in vitro IRBP-specific lymphocyte proliferation (B) and IFN-γ and IL-17 (C, D) production showed no significant difference among PBS treated mice, rAAV2-CMV-mIL-27p28 and rAAV2-CMV-GFP treated mice (p>0.05). Results are presented as mean ± standard deviation. (n = 5).

To evaluate the impact of rAAV2-CMV-mIL-27p28 subretinal injection on the immune system, we measured DTH in vivo, lymphocyte proliferation and production of IL-17 and IFN-γ in vitro. There was no difference concerning the DTH response among mice receiving PBS subretinal injection, mice receiving rAAV2-CMV-mIL-27p28 and mice receiving rAAV2-CMV-GFP subretinal injection (Figure 5A).

A similar lymphocyte proliferation upon stimulation with IRBP_161–180_ peptide was observed in both rAAV2-CMV-mIL-27p28 treated mice and rAAV2-CMV-GFP treated mice as compared with PBS treated controls. ConA could significantly lead to a much stronger lymphocyte proliferation as compared to IRBP_161–180_ peptide in these three groups. There was no difference concerning lymphocyte proliferation among PBS treated mice, rAAV2-CMV-mIL-27p28 treated mice and rAAV2-CMV-GFP treated mice (Figure 5B). Similarly the production of both IFN-γ and IL-17 by the lymphocytes from mice immunized with IRBP_161–180_ peptide and CFA was the same with that from PBS treated controls upon stimulation either with IRBP_161–180_ peptide or with ConA. ConA had a much stronger stimulation on the production of both cytokines as compared with IRBP_161–180_ peptide. There was no difference concerning the production of both cytokines among PBS treated mice, rAAV2-CMV-mIL-27p28 treated mice and rAAV2-CMV-GFP treated mice (Figure 5C, D). Lymphocytes from the tested three groups did not show a detectable proliferation, IL-17 production or IFN-γ production when cultured with medium alone.

## Discussion

In this study we showed that subretinal injection of rAAV2-CMV-mIL-27p28 in the mouse resulted in a high and stable IL-27p28 expression for at least 9 months. The amelioration of EAU disease severity in B10RIII mice was found to be associated with a decreased IL-17 expression and an increased IL-10 expression in rAAV2-CMV-mIL-27p28 injected eyes.

As IL-27p28 may play an important role in the regulation of autoimmune inflammation, we aimed to investigate its possible therapeutical effects using EAU as a model and gene transfer as a method of drug delivery. We successfully prepared the recombinant viral vector encoding mIL-27p28, based on adeno-associated virus, which provides a good vehicle for long-term safe and stable gene transfer. The present result revealed a prolonged local intraocular expression of mIL-27p28 following subretinal injection of rAAV2-CMV-mIL-27p28. The expression of IL-27p28 was 10.076 ng/ml (mean) within the eye after 21 days injection with 0.5 µl rAAV2-CMV-IL-27p28 vector (8.7×10^11^ v.g./ml). Transduction efficiency was also analyzed by histochemistry and showed uniform low grade transfer throughout the retina with strongest expression in the RPE, photoreceptor and outer plexiform layers (data not shown). These findings confirmed earlier studies from our group showing that the use of this vector system results in a specific localization within the layers of RPE cells and photoreceptor cells and no detectable transfer to other organs [29]. It is likely that subretinal injection of rAAV2-CMV-mIL-27p28, does not interfere with systemic function, as partially demonstrated by its ineffectiveness in the systemic immune response including lymphocyte proliferation and production of relevant cytokines.

For the purpose of examining the effect of IL-27p28 on EAU, we induced a clinically obvious intraocular inflammation in B10RIII mice by immunization with IRBP_161–180_ peptide and CFA. Subretinal injection of rAAV2-CMV-mIL-27p28 significantly attenuated the EAU activity, both clinically and pathologically. The fact that it not only blocked posterior uveitis but also inhibited anterior segment inflammation can be explained by the fact that the secreted IL-27p28 protein could diffuse from the posterior segment towards the front of the eye with the natural fluid flow [30]. As IFN-γ and IL-17 play an important role in the pathogenesis of EAU and IL-10 has a negative regulatory effect [31]–[32], we further investigated whether the inhibitory effect of subretinal injection of rAAV2-CMV-mIL-27p28 on this model was mediated by these cytokines. Induction of EAU was associated with a markedly increased intraocular expression of IFN-γ and IL-17 at the peak of inflammation. Subretinal injection of rAAV2-CMV-mIL-27p28 significantly inhibited the IL-17 expression but did not affect IFN-γ expression. It has been shown that IL27 promotes Th1 polarization of CD4^+^ lymphocytes though activation of STAT1 and the expression of T-bet [33]. On the other hand it also induces Tr1 regulatory lymphocytes that can in turn limit the Th1 response [33]. Previous studies have shown that IL-27 not only induces Th1 lymphocyte differentiation, but can at the same time suppress them, preventing excessive inflammation [8]–[10]. These findings may explain why we were not able to detect a change in the Th1 response in our model. Furthermore, subretinal IL-27p28 gene transfer caused a twofold increase in the ocular IL-10 expression during the clinical EAU episode as compared to controls receiving the GFP vector. Although the subretinal IL-27p28 gene transfer had a marked effect on the intraocular inflammation, we were not able to detect an effect on systemic immune response parameters including DTH, lymphocyte proliferation or production of relevant cytokines. This indicates that subretinal rAAV2-CMV-mIL-27p28 does not affect the afferent phase of EAU induction but exerts its inhibitory effect during the effector phase by local modulation of IL-17 and IL-10 production. Further confirmation is needed to show that IL27p28 transfection of retinal cells really blocks TH17 cell function. Experiments using IL27p28 transfected retinal explants co-cultured with T cells would be necessary to prove this.

As the EAU model used in this study is self-healing and has a short duration, it is difficult to study the effect of rAAV2-CMV-mIL-27p28 on an established intraocular inflammation. This issue could be addressed by studying the effect of IL-27p28 gene transfer on a chronic relapsing model of uveitis [34].

IL-27 is a heterodimer normally composed of two subunits namely Ebi3 and IL-27p28, whereby the Ebi3 subunit has been shown to be involved in the proper signaling and secretion of IL-27p28 [6], [35]. We only transfected the latter subunit and it would be interesting to investigate whether the use of the heterodimer would have a more pronounced effect on the EAU disease course and its associated intraocular cytokine response. In conclusion, we showed that subretinal injection of a rAAV2-CMV-mIL-27p28 construct achieved detectable and prolonged expression of mIL-27p28 protein within the eye and was able to control the severity of EAU. The inhibitory effect was attributed not only to the abundant mIL-27p28 secretion but also to the down-regulated of IL-17 expression and up-regulated IL-10 expression.

## Materials and Methods

### Ethics Statement

All experimental protocols were developed according to the Ethics Committee of the First Affiliated Hospital of Chongqing Medical University, Chongqing, China (Permit Number: 2009-201009) and in accordance with the Association for Research in Vision and Ophthalmology (ARVO) statement for the use of animals in Ophthalmic and Vision Research.

### Animals and Reagents

B10RIII mice were purchased from Jackson Laboratory (Bar Harbor, ME). All animals were housed under specific pathogen free conditions. Human interphotoreceptor retinoid binding protein peptide spanning amino acid residues 161–180 (IRBP_161–180_, SGIPYIISYLHPGNTILHVD) was synthesized by Shanghai Sangon Biological Engineering Technology & Services Ltd. Co. Complete Freund’s Adjuvant (CFA) containing 1.0 mg/ml mycobacterium tuberculosis (H37RA, ATCC 25177) was purchased from Sigma-Aldrich (St. Louis, MO).

### Vectors

The recombinant adeno-associated virus vector,rAAV2-CMV-mIL-27p28 (mouse IL-27p28, GenBank no. BC119402.1) was prepared as follows. Total mRNA was extracted from mouse splenocytes using RNeasy Plus Mini Kit (QIAGEN, Valencia, CA) and first-strand cDNA was synthesized with the Superscript III Reverse Transcriptase system (Invitrogen, Carlsbad, CA, USA). The coding sequence of mIL-27p28 was amplified using the following primers: forward, 5′ GGGGGTACCATGGGCCAGGTGACAGGA 3′ and reverse, 5′ TCTGTCGACTTAGGAATCCCAGGCTGAG 3′. The PCR product was inserted into T vector and T-mIL-27p28 was verified by DNA sequencing on the Applied Biosystems Model 3730 DNA Sequencing System (Invitrogen Biotechnology Co., Shanghai, China). The mIL-27p28 coding sequence was cut from T- mIL-27p28 with *Kpn*I and *Sal*I, and then subcloned into an AAV2-CMV backbone between the sites of *Kpn*I and *Sal*I. After sequence verification, large-scale production and purification of the vector was performed by Vector Gene Technology Company Ltd. Vector rAAV2-CMV-GFP was used as a control. The titers of vector batches were 8.7×10^11^ v.g./ml for rAAV2-CMV-mIL-27p28 and 1×10^12^ v.g./ml for rAAV2-CMV-GFP, respectively.

### Subretinal Injection

The pupil of mice was dilated with 1% atropine eyedrops following general anesthesia with an intraperitoneal injection of pentobarbital (0.1 g/kg). Subretinal injection was performed under sterile conditions according to the method reported previously [36]. A 30-gauge needle was use to make an aperture within the pupil area through the cornea. A blunt 33-gauge needle was carefully inserted through the corneal aperture avoiding damage to the lens and penetrating the neuroretina. With the aid of a micro-injection system, 0.5 µl of vector was injected into the subretinal space in 30 seconds. The sign of retinal blebs indicated a successful delivery. Antibiotic ointment was used daily for three days following subretinal injection. Successfully injected mice without surgical complications including temporal corneal edema, anterior or posterior synechiae, cataract and vitreous or retinal hemorrhage were used for further study as described below.

### Intraocular Cytokine Immunoassay

Mice only receiving a subretinal injection of rAAV2-CMV-mIL-27p28 or rAAV2-CMV-GFP were sacrificed at various time points. Serum were collected for assay of IL-27p28. Normal mice and another group of mice receiving a subretinal injection and a subsequent immunization with IRBP_161–180_ peptide and CFA were sacrificed on day 12 and day 21 post-immunization. For ocular fluid sample preparation, the injected eye and contralateral eye were enucleated. The conjunctival and optic tissues were carefully removed and the globes were briefly sonicated with homogenizing solution(20% glycerol, 10 mM KCl, 2 mM MgCl2, 0.1% Triton, 300 mM NaCl, 0.5 mM dithiothreitol, 20 mM HEPES and Anti-protease Complete TM cocktail in H2O, 10 µl/mg). After centrifugation at 11000×*g* for 5 min, supernatants from homogenized eyes were collected. Concentrations of IL-27p28, IL-17, IL-10 and IFN-γ were determined using commercially available ELISA kits according to the manufacturer’s directions (R&D System, Minneapolis, MN) with a detection limit of 15.6 pg/ml, 15.6 pg/ml, 31.3 pg/ml and 31.3 pg/ml respectively.

### Induction and Clinical Assessment of EAU

Human IRBP_161–180_ peptide was solubilized in PBS and emulsified 1∶1 v/v in complete Freund’s adjuvant (CFA) supplemented with 1.0 mg/ml *mycobacterium tuberculosis* strain (MTB). A total of 200 µl emulsion containing 50 µg human IRBP_161–180_ peptide was given subcutaneously at the base of the tail and both thighs for each mouse. Clinically visible EAU was evidenced by conjunctival hyperemia, ciliary injection, corneal edema, aqueous flare and cells, and posterior synechiae. Clinical scores of EAU were recorded by slit lamp microscopy on day 12 after immunization. The clinical severity of ocular inflammation was scored on a scale of 0–5 in half-point increments in a masked manner, based on five separate criteria described previously [29].

### Histopathology

Eyes were enucleated on day 14 after immunization and fixed in 4% buffered formaldehyde for 1 hour at room temperature. Tissues were embedded in paraffin. A 4–6 µm vertical serial section was cut through the papillary-optic nerve axis and stained by haematoxylin and eosin. The intensity of EAU was graded by light microscopy in a masked fashion on a scale of 0 to 4, as described earlier [37].

### Assay for Delayed Type Hypersensitivity

Nineteen days after immunization the mice were anesthetized by intraperitoneal injection of pentobarbital (0.1 g/kg) and ear thickness of both ears measured with a micrometer. Human IRBP_161–180_ peptide (10 µg) in 10 µl PBS was injected into the right pinna of each mouse, whereas 10 µl PBS was injected into the left pinna as a control. Forty-eight hours later, the mice were anesthetized with pentobarbital and ear thickness was measured again. Specific ear swelling was calculated as the difference in thickness (in µm) of the right ear minus the difference in thickness (in µm) of the left ear.

### IRBP-specific Lymphocyte Responses

The spleen and draining lymph nodes were collected from immunized mice on day 21. A single cell suspension was prepared. Cells (2×10^6^ cells/ml) were cultured in a 96-well plate in triplicate with RPMI 1640 medium (Gibco, Grand Island, NY, USA) containing 2 mM L-glutamine, 5×10-5 M2-ME, 1 mM sodium pyruvate, 0.1 Mm nonessential amino acids and 10% FBS and challenged with 10 µg/ml IRBP161–180, 1 µg/ml Concanavalin A (Sigma) or medium alone for 72 hours. The lymphoproliferative response was detected by a modified MTT assay using a cell counting kit (Cell Counting Kit-8; Sigma) as described previously [38]. IL-17 and IFN-γ concentrations in the supernatants were measured using commercially available ELISA kits according to the manufacturer’s directions (R&D System, Minneapolis, MN) with a detection limit of 15.6 pg/ml and 31.3 pg/ml respectively.

### Statistical Analysis

Data are expressed as mean ± standard deviation (SD). Severity of EAU was analyzed using the Kruskal-Wallis test followed by the Mann-Whitney U test. Expression of cytokines within the eyes was analyzed using the Paired T test and Mann-Whitney U test. DTH, lymphocyte proliferation and cytokine production were analyzed using ANOVA. A p value below 0.05 was considered to be significantly different. All experiments were repeated at least twice.
